# Bioenergetic effects of hydrogen sulfide suppress soluble Flt-1 and soluble endoglin in cystathionine gamma-lyase compromised endothelial cells

**DOI:** 10.1038/s41598-020-72371-2

**Published:** 2020-09-25

**Authors:** Lissette Carolina Sanchez-Aranguren, Shakil Ahmad, Irundika H. K. Dias, Faisal A. Alzahrani, Homira Rezai, Keqing Wang, Asif Ahmed

**Affiliations:** 1 Aston Medical Research Institute, Aston Medical School, Birmingham, UK; 2 Mirzyme Therapeutics, Innovation Birmingham Campus, Faraday Wharf, Holt Street, Birmingham, B7 4BB UK; 3 Department of Biochemistry, ESC Research Unit, Faculty of Science, King Fahd Medical Research Center, King Abdulaziz University, Jeddah, 21589 Saudi Arabia

**Keywords:** Pre-eclampsia, Medical research

## Abstract

Endothelial dysfunction is a hallmark of preeclampsia, a life-threatening complication of pregnancy characterised by hypertension and elevated soluble Fms-Like Tyrosine Kinase-1 (sFlt-1). Dysregulation of hydrogen sulfide (H_2_S) by inhibition of cystathionine γ-lyase (CSE) increases sFlt-1 and soluble endoglin (sEng) release. We explored whether compromise in CSE/H_2_S pathway is linked to dysregulation of the mitochondrial bioenergetics and oxidative status. We investigated whether these effects were linked to CSE-induced sFlt-1 and sEng production in endothelial cells. Here, we demonstrate that CSE/H_2_S pathway sustain endothelial mitochondrial bioenergetics and loss of CSE increases the production of mitochondrial-specific superoxide. As a compensatory effect, low CSE environment enhances the reliance on glycolysis. The mitochondrial-targeted H_2_S donor, AP39, suppressed the antiangiogenic response and restored the mitochondrial bioenergetics in endothelial cells. AP39 revealed that upregulation of sFlt-1, but not sEng, is independent of the mitochondrial H_2_S metabolising enzyme, SQR. These data provide new insights into the molecular mechanisms for antiangiogenic upregulation in a mitochondrial-driven environment. Targeting H_2_S to the mitochondria may be of therapeutic benefit in the prevention of endothelial dysfunction associated with preeclampsia.

## Introduction

Oxidative stress and elevated antiangiogenic growth factors^1–3^ are recognised markers of preeclampsia. This complication of pregnancy is characterised by maternal endothelial dysfunction^4^, which is also a hallmark of cardiovascular disease. Circulating levels of antiangiogenic factors, such as soluble Fms-Like Tyrosine Kinase-1 (sFlt-1) and soluble endoglin (sEng) increase weeks before the onset of preeclampsia^3,5,6^. Soluble Flt-1 appears to be responsible fo the maternal widespread endothelial dysfunction^5,7,8^ that characterises this disorder. High levels of sFlt-1 in preeclampsia is responsible for suppressing angiogenesis and removal of this culprit protein restores it^9^. A number of splice variants of sFlt-1 have been identified^8,10^. In particular, the expression of the sFlt-1 i13 variant is widely distributed in most human tissues and it is significantly elevated in preeclamptic women (> 34 weeks)^11^ while the sFLT-1 e15a variant, highly expressed in the placenta, is significantly upregulated in severe preeclampsia^12^.

Wang and colleagues demonstrated that the inhibition of cystathionine-γ-lyase (CSE) results in the increased production of sFlt-1 and sEng in vivo^13^. The CSE is the main hydrogen sulfide (H_2_S) producing enzyme in the endothelium^14^ and to a lesser extent H_2_S is produced by cystathionine β-synthetase (CBS) and 3-mercaptopyruvate sulfurtransferase (MST)^15,16^. H_2_S is a gaseous signalling molecule with recognised anti-inflammatory^17^ and cytoprotective^18,19^ properties. Dysregulation in H_2_S has been identified in preeclampsia^13^ as well as in low-grade chronic inflammatory conditions such as diabetes^20^ and obesity^21^, which are risk factors for preeclampsia. The administration of H_2_S donors have shown to suppress sFlt-1 and sEng in endothelial cells, and to restore fetal growth in mice treated with the CSE inhibitor, DL-propargylglycin^13^. These observations denote that CSE/H_2_S system acts as a protective pathway required for healthy pregnancy and acts as a brake on regulating the levels of sFlt-1 and sEng. Defect in CSE/H_2_S pathway contributes to preeclampsia by releasing this brake and supports the Ahmed’s Protective Law of preeclampsia^22^.

Recently, we demonstrated that sFlt-1 in a dose-dependent manner inhibited the mitochondrial respiration and promoted mitochondrial-specific superoxide production in endothelial cells^23^. This suggests an association between antiangiogenic factors and mitochondrial dysfunction. Others have shown that chronic infusion of recombinant sFlt-1 on a daily basis for eight days to pregnant rats led to increased vascular superoxide production^24^ and exacerbated oxidative stress, provoked mitochondrial swelling and induced apoptosis in trophoblasts in sFlt-1-injected mice^25^. These observations suggest that high circulating sFlt-1 contributes to vascular dysfunction by modulating the mitochondrial integrity in endothelial cells and placenta.

We sought to explore whether a compromise in the CSE/H_2_S pathway is linked to dysregulation of the mitochondrial bioenergetics and oxidative stress and to investigate if these effects are linked to sFlt-1 and sEng production in CSE compromised human umbilical vein endothelial cells (HUVEC). Our findings provide evidence to show that CSE-derived H_2_S in the mitochondrion acts to suppress antiangiogenic factors. Loss of CSE increased oxidative status in endothelial cells, while treatment with the mitochondrial-targeted H_2_S donor, AP39 promoted the mitochondrial bioenergetics and suppressed sFlt-1 and sEng production. AP39 revealed that upregulation of sFlt-1, but not sEng is independent of the mitochondrial H_2_S metabolising enzyme, sulfide quinone oxidoreductase (SQR). These results suggest that therapeutically targeting H_2_S to the mitochondria should be considered in the management of endothelial dysfunction associated with preeclampsia.

## Results

### Downregulation of endothelial CSE results in reactive oxygen species generation

**Figure 1:**
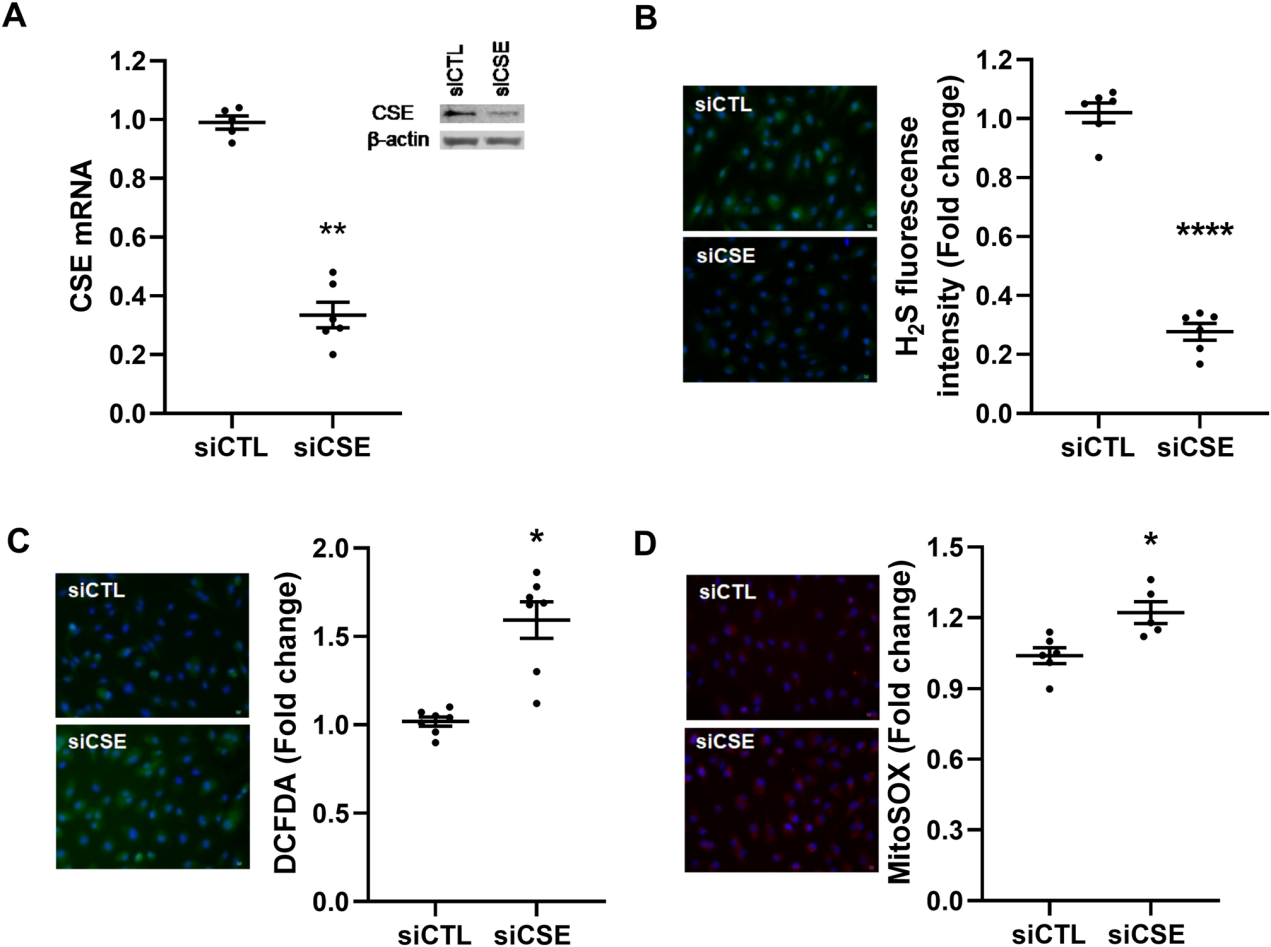
Compromise in endothelial CSE by siRNA silencing enhances oxidative status in HUVEC. **(A)** CSE gene and protein expression were determined by RT-PCR and Western blot, respectively in CSE and control siRNA transfected HUVEC. Statistical significance was measured via Mann–Whitney test. **(B)** Intracellular H_2_S availability was measured by fluorescence microscopy using the specific fluorescence probe SF7-AM. Statistical significance was measured via unpaired T test. **(C)** Intracellular reactive oxygen species were detected by fluorescence microscopy using the fluorescent probe DCFDA. Statistical significance was measured via Mann–Whitney test. **(D)** Mitochondrial superoxide was measured by fluorescent microscopy using MitoSOX Red probe. Statistical significance was measured via unpaired T test. A shows a representative Western blot from n = 1 experiment. As indicated in the materials and methods section, Western blot membranes were cropped for consise display. Microscopy images were taken using the 20X objective lens ans indicated in the materials and methods section. Values are expressed as means ± SEM and each dot represents a biological replicate examined in three independent experiments. n = 3–6 experimental replicates. *P < 0.05, **P < 0.01, ***P < 0.001 ****P < 0.0001 vs siCTL. Figure was created using GraphPad Prism 8.1 (GraphPad Software, La Jolla, CA; https://www.graphpad.com/scientific-software/prism/).

Endogenous H_2_S bioavailability has been proposed to regulate energy production in mammalian cells under increased energetics demands^26^. To study whether endogenous CSE-dependant H_2_S has a role in the regulation of endothelial cells bioenergetics, we inhibited CSE mRNA levels by siRNA transfection in HUVEC and evaluated the mitochondrial function and cellular oxidative status. Effective knockdown of CSE was confirmed by qPCR and Western blot (Fig. 1A and Supplementary Fig. 1a), non-silencing siRNA was used as control (siCTL). Consistently, the bioavailability of H_2_S was reduced in siCSE transfected HUVEC as examined by microscopy using the fluorescent probe sulfidefluor-7 acetoxymethyl ester (SF7-AM) when compared to siCTL (*p* < 0.0001) (Fig. 1B).

Since mitochondrial dysfunction is associated with increased production of reactive oxygen species, we measured cellular superoxide production and mitochondrial-specific superoxide production using the fluorescent probe DCFDA and MitoSOX Red, respectively. Cellular reactive oxygen species (ROS) levels increased 50% in CSE silenced-HUVEC (*p* = 0.01) (Fig. 1C) while the production of specific mitochondrial superoxide was increased by 25% when compared to control (*p* = 0.04) (Fig. 1D).

### Downregulation of endothelial CSE causes perturbation in bioenergetics

**Figure 2:**
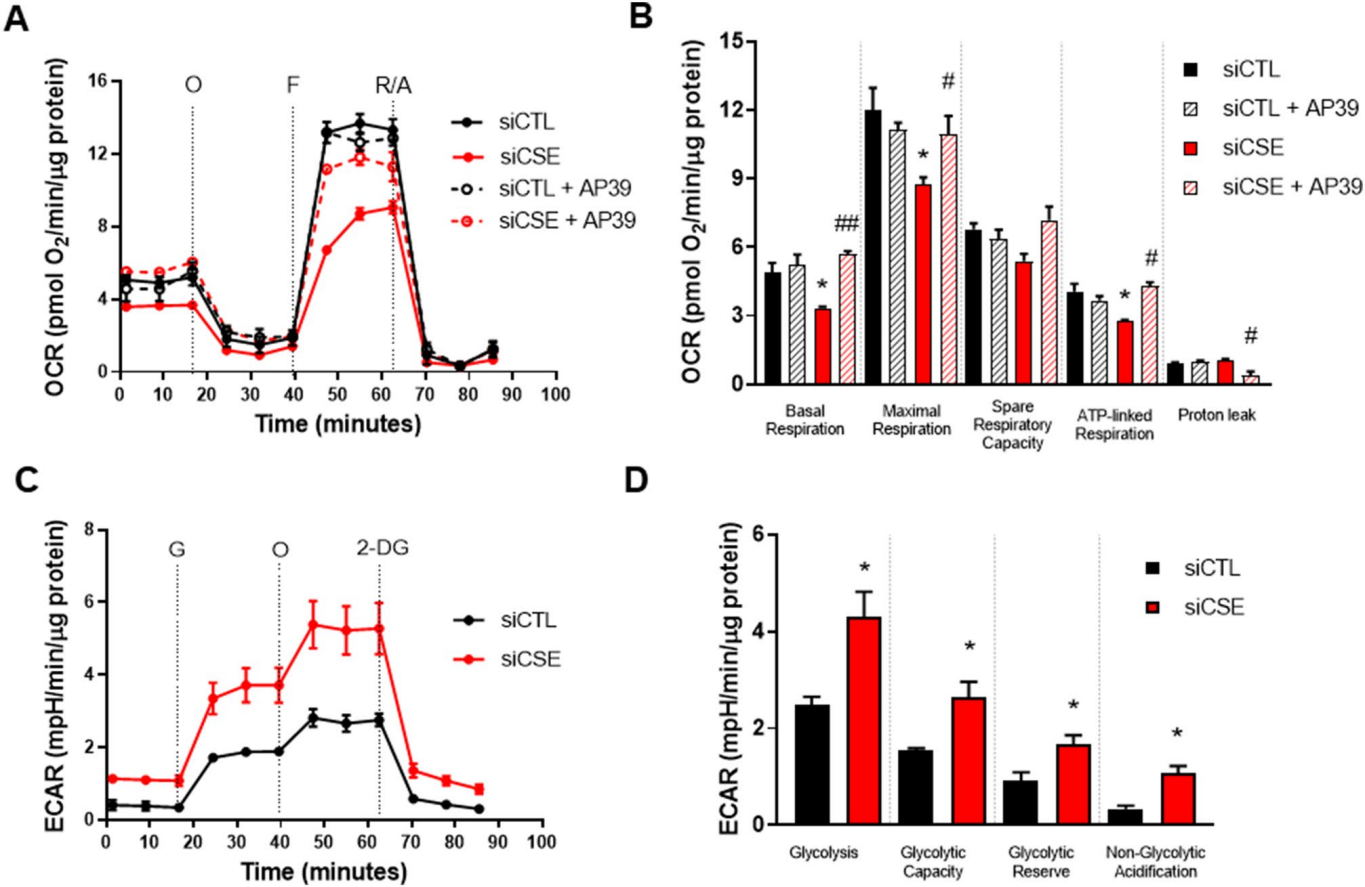
Inhibition of endothelial CSE perturbates the mitochondrial bioenergetics and enhances glycolysis. **(A)** Traces of oxygen consumption rates (OCR) and **(B)** parameters of mitochondrial function (basal, maximal, spare respiratory capacity, ATP-linked respiration, and proton leak) were calculated after sequential injections of oligomycin (O) (1 mM), carbonylcyanide-p-trifluoromethoxyphenylhydrazone (FCCP) (0.5 μM) and a mixture of rotenone and antimycin A (R/A) (1 mM). Statistical significance was measured via ordinary one-way ANOVA and Bonferroni’s post-hoc test. **(C)** Traces of extracellular acidification rates (ECAR) were measured after sequential injections of glucose (G) (10 mM), oligomycin (O) (1 mM), and 2-Deoxiglucose (2-DG). **(D)** Parameters of the glycolytic pathway: glycolysis, glycolytic capacity, glycolytic reserve, and non-glycolytic acidification were calculated from ECAR measurements using a Seahorse XF24 Analyser. Statistical significance was measured via unpaired T test. Values are expressed as means ± SEM, n = 3–6 experimental replicates. *P < 0.05 vs siCTL. ^#^*P* < 0.05, ^##^*P* < 0.01 vs siCSE. Figure was created using GraphPad Prism 8.1 (GraphPad Software, La Jolla, CA; https://www.graphpad.com/scientific-software/prism/).

Previous studies have indicated metabolic disturbances in the absence of CSE activity^27,28^. We sought to investigate whether the loss of CSE might be associated with alterations in the overall cellular bioenergetics. First, we evaluated the effect of the loss of CSE/H_2_S pathway over the function of mitochondria by monitoring oxygen consumption rates (OCR) using a Seahorse Agilent XF24 Analyser. When CSE is compromised, the overall parameters of mitochondrial function are suppressed (Fig. 2A). SiRNA-mediated silencing of CSE significantly reduced the basal (*p* = 0.02), maximal (*p* = 0.02) and ATP-linked OCR (*p* = 0.04) in comparison to control. The spare respiratory capacity and OCR-linked to proton leak were reduced but not statistically different from siCTL (*p* = 0.19 and *p* = 0.94, respectively).

To confirm the role of mitochondrial H_2_S in promoting HUVEC bioenergetics, cells were treated with the mitochondrial-targeted H_2_S donor, AP39.. AP39 protects against oxidative damage by abrogating mitochondrial specific ROS and improving the mitochondrial bioenergetics in endothelial cells^29^.HUVEC were treated with increasing concentrations of AP39 (10–1,000) nM and cell viability was detected by MTT. We determined that 100 nM of AP39 was the optimal concentration to use in further experiments (Supplementary Fig. 2). Control cells treated with AP39 showed no statistical difference in the parameters of mitochondrial function when compared to siCTL. AP39 improved the basal (*p* = 0.008), maximal (*p* = 0.04) and ATP-linked OCR (p = 0.01) in siCSE-transfected HUVEC in comparison to siCSE only suggesting that AP39 acts to enhance the mitochondrial function. The spare respiratory capacity was improved, however, not significantly different from siCSE-transfected cells (*p* = 0.11). Proton leak linked-OCR was reduced in siCSE transfected HUVEC treated with AP39 (*p* = 0.01) when compared to siCSE only (Fig. 2B).

### Inhibition of CSE enhances the glycolytic capacity in HUVEC

Once we assessed that loss of CSE activity in HUVEC leads to signs of mitochondrial dysfunction, we evaluated the overall effect on the glycolytic pathway by measuring the extracellular acidification rates (ECAR) using a Seahorse XF24 Analyser. Our results show that when CSE is compromised, rates of extracellular acidification are significantly increased when compared to control (Fig. 2C). Overall parameters of the glycolytic pathway were measured after sequential injections of glucose, oligomycin, and 2-DG. The glycolysis rate (*p* = 0.02), glycolytic capacity (*p* = 0.03), glycolytic reserve (*p* = 0.03), and non-glycolytic acidification (*p* = 0.01) were significantly higher in siCSE-transfected HUVEC when compared to control (Fig. 2D). These results suggest that reduced bioavailability of CSE-derived H_2_S impair the overall cellular bioenergetics allowing an increased reliance on the less efficient glycolytic pathway possibly to sustain energetic demands not met by oxidative phosphorylation (OXPHOS)^23,30,31^.

### Role of mitochondrial H_2_S in suppressing antiangiogenic factors

Previous observations in our laboratory demonstrated that when CSE is compromised either in endothelial cells or in vivo, levels of sFlt-1 and sEng are significantly increased^13^. Furthermore, administration of H_2_S donors under these conditions cause a reduction in the levels of these antiangiogenic factors^13^. Therefore, we sought to evaluate whether these events happen in a mitochondrial-driven environment. To that end, we forced mitochondrial metabolism by replacing glucose with galactose in the growth media (Supplementary Fig. 3) to demonstrate whether the mitochodnria play a pivotal role in the regulation of sFlt-1 and sEng when CSE is compromised. In other scenarios, it has been shown that cells grown in galactose as a sole carbohydrate source, display a more aerobic state mainly because of a reduction in ATP production via glycolysis and an increase mitochondrial oxidative capacity^32–34^. Using MTT assay, we determined that the replacement of glucose with galactose does not affect the cell viability (Supplementary Fig. 4).

**Figure 3:**
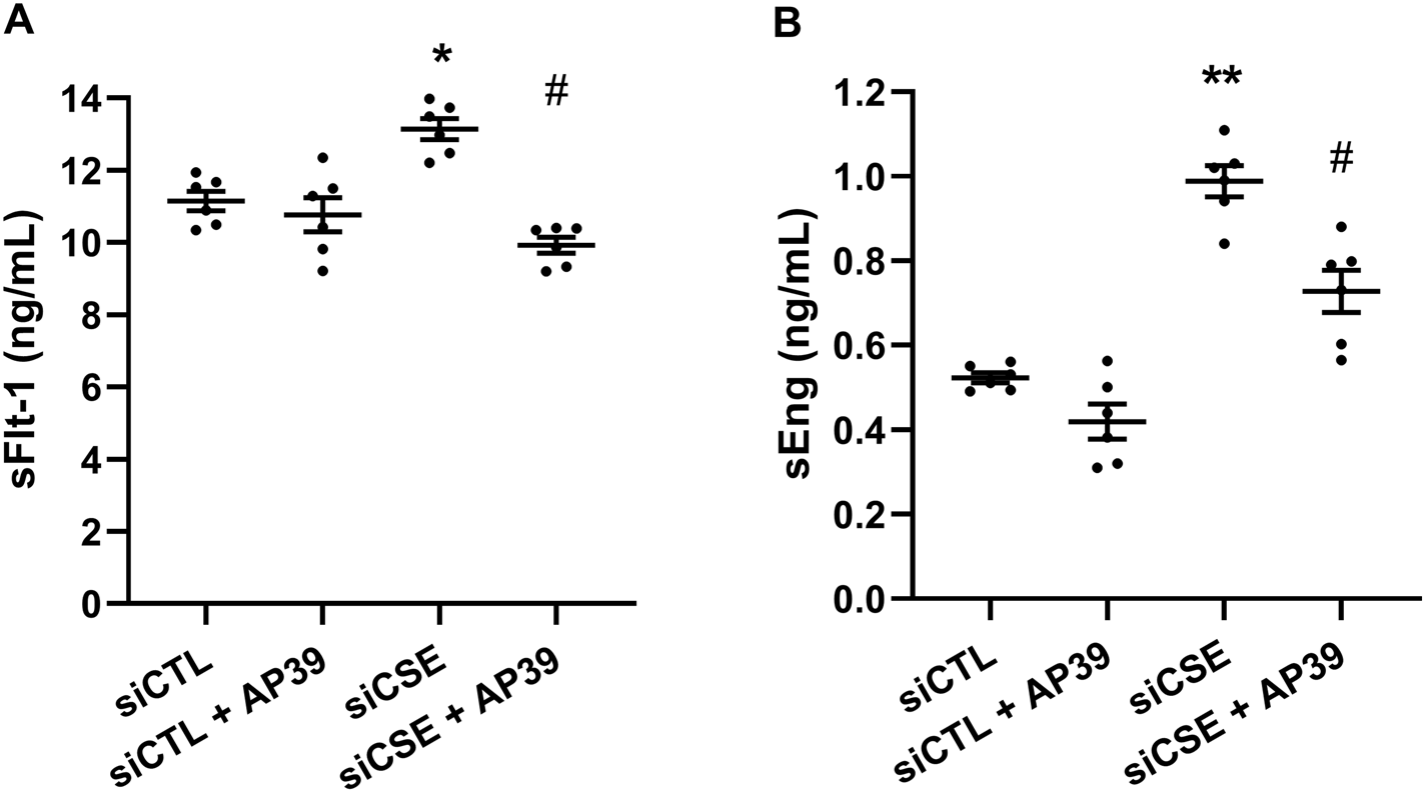
Mitochondrial H_2_S regulates sFlt-1 and sEng production in HUVEC. HUVEC transfected with CSE siRNA were cultured in galactose supplemented media. **(A)** of sFlt-1 and **(B)** sEng were measured in culture media by ELISA. Values are expressed as means ± SEM and each dot represents a biological replicate examined in three independent experiments. n = 3–6 experimental replicates. Statistical significance was measured via one-way ANOVA and Bonferroni’s post-hoc test. *P < 0.05, **P < 0.01 vs siCTL, ^#^*P* < 0.05 vs siCSE. Figure was created using GraphPad Prism 8.1 (GraphPad Software, La Jolla, CA; https://www.graphpad.com/scientific-software/prism/).

**Figure 4:**
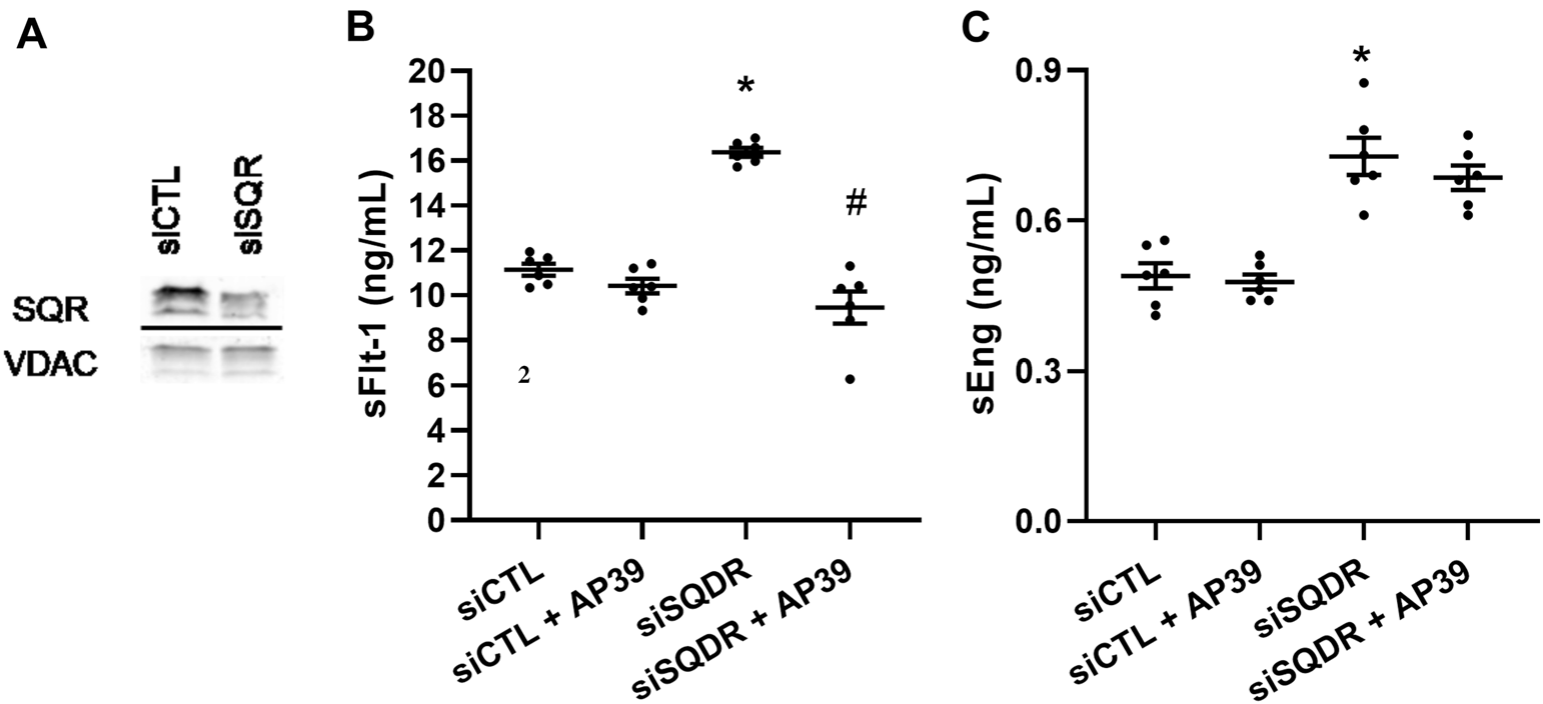
Role of SQR in the regulation of antiangiogenic factors in HUVEC. **(A)** HUVEC were transfected with SQR siRNA and SQR protein expression was evaluated by Western blot using specific antisera and VDAC protein expression was used as control. siSQR transfected HUVEC were exposed to AP39 (100 nM) for 24 h and **(B)** levels of sFlt-1 and **(C)** sEng were measured in culture media by ELISA. A shows a representative Western blot from n = 1 experiment. As indicated in the materials and methods section, Western blot membranes were cropped using Image J software for consise display. A delineating line was placed between cropped blot images. Statistical significance was measured via one-way ANOVA and Bonferroni’s post-hoc test in **(B)** and Kruskal Wallis and Dunn’s post-hoc test in **(C)**. Values are expressed as means ± SEM and each dot represents a biological replicate examined in three independent experiments. n = 3–6 experimental replicates. *P < 0.05 vs siCTL, #*P* < 0.05 vs siSQR. Figure was created using GraphPad Prism 8.1 (GraphPad Software, La Jolla, CA; https://www.graphpad.com/scientific-software/prism/).

We exposed siCSE silenced HUVEC to galactose media and levels of sFlt-1 were measured by ELISA. Interestingly, our results showed that inhibition of siCSE significantly increased sFlt-1 levels when compared to control (*p* = 0.04). Treatment with AP39 showed that restoration of mitochondrial H_2_S reduced sFlt-1 production in siCSE silenced HUVEC, when compared to siCSE-transfected HUVEC (*p* = 0.034) (Fig. 3A).

Using the same approach, we evidenced that when OXPHOS is forced in CSE-compromised cells, there is a significant increase in the production of sEng (*p* = 0.01). Interestingly, treatment with AP39, reduced the production of sEng (*p* = 0.014) (Fig. 3B). Similar results were obtained in CSE-compromised HUVEC treated with the mitochondrial ROS scavenger, mitoTempo (Supplementary Fig. 5). Our metabolic-restriction model using galactose to enhance the mitochondrial reliance of HUVEC, allowed to evidence a link between mitochondria and CSE/H_2_S deficient pathways in the regulation of antiangiogenic molecules.

**Figure 5:**
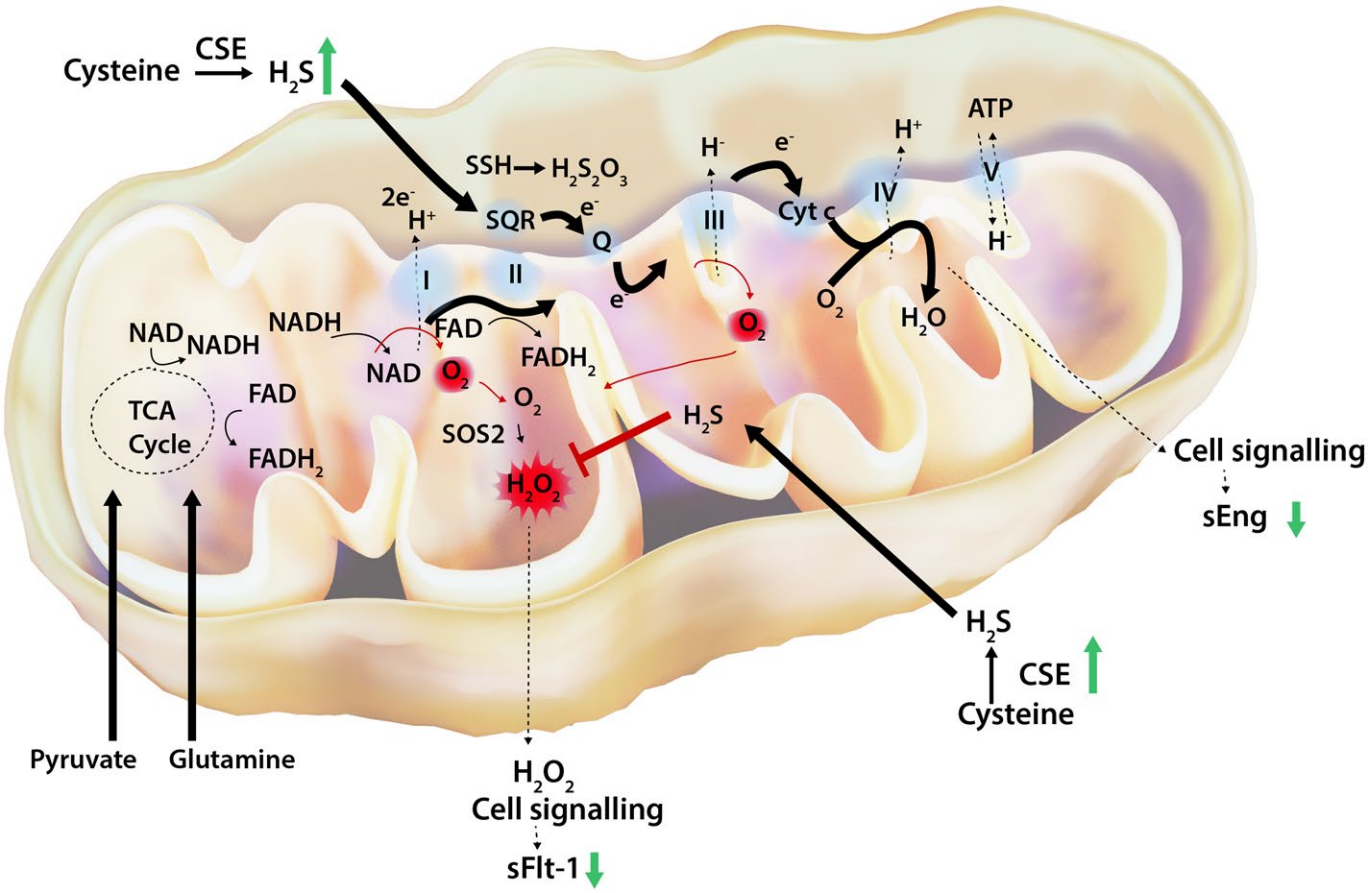
The proposed molecular mechanism of mitochondrial H_2_S-mediated regulation of antiangiogenic factors, sFlt-1 and sEng in endothelial cells. CSE-derived H_2_S diffuse to the mitochondrion and donates e^-^ to the membrane-associated sulfide quinone oxidoreductase (SQR) that couples the oxidation of H_2_S into thiosulfate (H_2_S_2_O_3_) while transferring e^-^ to coenzyme Q (Q) in the electron transport chain (ETC). Mitochondrial metabolism of H_2_S in SQR sustains mitochondrial respiration by accelerating the consumption of oxygen (O_2_) at complex IV and therefore limiting the production of superoxide (O_2_^.-^) at complexes I–III. Concomitantly, oxidation of carbon-based substrates (pyruvate and glutamine) leads to the reduction of the NAD or FAD coenzymes to NADH and FADH_2_, respectively yielding e^-^ to Q and promoting mitochondrial respiration. O_2_^.-^ is metabolised to hydrogen peroxide (H_2_O_2_) by SOD2. Also, H_2_S acts as a ROS scavenger. We propose that H_2_S abrogates H_2_O_2_ production, resulting in the regulation of soluble Flt-1 (sFlt-1) release. H_2_S enhances the electron transport chain through SQR-dependant mechanisms that results in modulation of soluble endoglin (sEng) levels. Thickness of the arrows proportional to the underlying flux. Putative pathways linking H_2_S with sFlt-1 and sEng release are represented by dashed lines. This diagram was drawn using GIMP 2.1 software.

### Metabolism of H_2_S in mitochondrial SQR regulate sEng but not sFlt-1 production in HUVEC

Finally, to elucidate the role of mitochondrial H_2_S-metabolising enzyme, SQR in the regulation of sFlt-1 and sEng production, we silenced SQR using siRNA in HUVEC cultured in standard conditions. Effective SQR silencing was evidenced by WB using SQR antisera and VDAC as control (Fig. 4A and Supplementary Fig. 1b).

Inhibition of SQR resulted in a significant upregulation of sFlt-1 production in HUVEC, compared to siCTL (p = 0.012). The administration of AP39 to these experiments showed that in the absence of H_2_S metabolising enzyme, SQR, levels of sFlt-1 are significantly supressed (*p* = 0.02). These results suggest that loss of SQR, by impairing OXPHOS, might result in increased mitochondrial ROS limited by AP39 (Fig. 4B).

In contrast, inhibition of SQR resulted in a significant increase of sEng production in HUVEC when compared to control (*p* = 0.02). However, the administration of AP39 did not suppress sEng production when SQR was compromised, when compared to siSQR HUVEC (*p* = 0.87) (Fig. 4C). These observations suggest that the metabolism of H_2_S in mitochondrial SQR is necessary for sEng regulation but not sFlt-1 in HUVEC. Our observations propose that H_2_S bioavailability, when restored within the mitochondria, regulate antiangiogenic factors production. Mitochondrial H_2_S may act either as a modulator of the cellular bioenergetics or as a mitochondrial oxidant scavenger in the regulation of endothelial oxidative stress (Fig. 5). Therefore, mitochondrial-specific H_2_S donors may be of therapeutic potential to regulate sFlt-1 and sEng production.

## Discussion

The risk associated with preeclampsia is not only restricted to the period of pregnancy. After pregnancy, women diagnosed with preeclampsia have twice the long-term risk of stroke or heart attack compared to women without this disorder^35^. The increase in circulating sFlt-1 that occurs during normal pregnancy compared to pre-pregnancy is many-fold greater than the difference between third trimester normal pregnancy and preeclampsia levels^2^. Our laboratory had proposed that preeclampsia is a consicuence of high sFlt-1 and low heme oxygenase-1 and/or CSE^1,22^. Here, we demonstrate that when endothelial CSE/H_2_S pathway is compromised, it perturbates the mitochondrial bioenergetics and increases the production of mitochondrial-specific ROS. These effects were accompanied by a concomitant reliance on glycolysis pathway, possibly to sustain energetic demands. Using a substrate restrictive approach, replacing glucose with galactose in the cells culture media, we demonstrate that the metabolism of mitochondrial specific H_2_S reduces sFlt-1 and sEng production in HUVEC. Our results provide novel insights into the mechanistic role of mitochondrial H_2_S in low CSE environment and suggests mitochondria as a new metabolic target in preeclampsia management.

Although endogenous H_2_S might be produced by different sources, such as CSE, CBS, and 3MST, endothelial CSE is the main H_2_S synthetizing enzyme in the vascular system^14^. In the present study, endothelial silencing of CSE resulted in a reduction of the mitochondrial activity and an increased reliance on the glycolytic pathway. Previous reports have shown that H_2_S is an inorganic substrate for the electron transport chain that enhances the bioenergetic role of organic substrate-derived electron (e^-^) donors (such as pyruvate and glutamine)^36,37^. In the electron transport chain, H_2_S diffuse to the mitochondrion and donates e^-^ to SQR coupling the oxidation of H_2_S into thiosulfate while transferring e^-^ to coenzyme Q^38^. Mitochondrial H_2_S sustains OXPHOS by accelerating the consumption of oxygen at complex IV^39^ and limiting the production of superoxide at complexes I–III^29^.Our results provide evidence that endothelial CSE-derived H_2_S is crucial to sustaining endothelial cellular bioenergetics. As previously reported, levels of L-cysteine are three times higher in the mitochondrial matrix and cytosolic CSE can translocate to the mitochondria to stimulate smooth muscle cells bioenergetics and ATP production^26^.

While previous studies have evidenced that H_2_S plays a key role in the regulation of cellular bioenergetics, compensation on other metabolic pathways when H_2_S is reduced had not been previously reported. In this study, mitochondrial H_2_S was found to positively regulate OXPHOS and loss of CSE/H_2_S pathway showed to promote mitochondrial-specific ROS production. As a consequence, an upregulation of the glycolytic pathway was evidenced, possibly to compensate for the lack of energetic drive through mitochondria. As reported before, CSE^-/-^ mice showed increased immunofluorescence staining for γH2AX, a marker for DNA oxidative stress on kidney sections when compared to wild-type mice^40^. Consistently, overexpression of CSE abrogated mitochondrial specific-ROS production induced by the mitochondrial complex III inhibitor, antimycin A in HEK293 cells^40^. In preeclampsia, the activation of the maternal endothelium by ROS results in endothelial dysfunction^4^. Therefore, our observations suggest that reduced bioavailability of endogenous H_2_S alters the redox balance, increases the oxidative status within the mitochondria and perturbates the overall cellular bioenergetics.

It has been suggested that the main function of mitochondria in endothelial cells relies on calcium homeostasis^41^. Extensive research also recognises their role in endothelial cells bioenergetics^42^. Normal endothelial cells rely mainly in glycolytic pathways. However, acetil-CoA derived from glucose-derived carbons, glutamine and fatty and amino acid metabolism sustain the Krebs cycle, driving the mitochondrial production of ATP in endothelial cells^43^. The effects of the reduced bioenergetics drive in CSE-compromised endothelial cells have not previously being addressed. Endothelial cells have metabolic plasticity that allows rapid conversion from a quiescent to a proliferating phenotype during angiogenesis^44^. Reliance on OXPHOS is far more efficient in the generation of ATP than glycolysis^45^. However, different stimuli can provoke a metabolic shift and promote glycolysis over OXPHOS, perhaps to maintain constant rates of ATP when the mitochondrial function is impaired^23,30,31^. Jarosz and colleagues, showed a link between H_2_S and metabolic reprogramming. They demonstrated that CSE and CBS-derived polysulfides inhibited glyceraldehyde 3-phosphate dehydrogenase, which is a key enzyme in the glycolytic pathway^46^. Consequently, endogenous H_2_S might be implicated in the regulation of energetic pathways. Previous studies have shown that wound healing and microvessel formation are impaired in CSE^-/-^ mice and these effects are not restored by Vascular Endothelial Growth Factor (VEGF) stimulus^47^. Our study shows that reduced H_2_S leads to oxidative stress and reduced mitochondrial activity while enhancing less efficient metabolic pathways. This suggesting a link between mitochondrial H_2_S and metabolic signalling and the modulation of the endothelial function by exogenous mitochondria H_2_S donors may restore function in conditions such as preeclampsia, where CSE is compromised.

Previous reports from our laboratory showed a direct link between defective CSE/H_2_S pathway and increased production of antiangiogenic factors^13^. In addition, we showed an association between high sFlt-1 and mitochondrial dysfunction^23^. We demonstrate that mitochondria play a key role in elevating the production of sFlt-1 and sEng in low CSE/H_2_S settings. As endothelial cells are recognised to rely mainly in glycolytic pathways^43^, we used a substrate-based approach (Supplementary Fig. 3)^32–34^, to force mitochondrial metabolism in CSE siRNA transfected HUVEC. This approach allowed us to evidence the mechanistic role of mitochondria in regulating sFlt-1 and sEng in dysregulated CSE/H_2_S. Our results showed that replacement of glucose with galactose does not affect the cells viability (Supplementary Fig. 4). We showed that the mitochondrial H_2_S donor AP39, significantly suppressed sFlt-1 and sEng production. Recent reports by Covarrubiasand colleagues showed that AP39 reversed the upregulation of sFlt-1 in hypoxia-treated primary trophoblasts^48^. Given that mitochondrial H_2_S has proven abilities to preserve the mitochondrial function in different disease models, our results propose that mitochondrial H_2_S metabolism may be linked to the production of sFlt-1 and sEng (Supplementary Fig. 5). Mitochondrial-targeted H_2_S donors offers a new therapeutic source in the management of endothelial dysfunction associated with preeclampsia. It is important to point out that our restrictive metabolic approach do not resemble normal metabolic culture conditions for endothelial cells and the scope of this evidence in vivo settings remain to be investigated.

As H_2_S can either enhance the electron transport chain or exert antioxidant effects within the mitochondrion^19,29,49,50^, we evaluated the effect of the mitochondrial metabolising enzyme, SQR in regulating antiangiogenic factors production in endothelial cells. SQR catalyses the transference of e^-^ to coenzyme Q from H_2_S with its subsequent oxidation into thiosulfate in the electron transport chain^51^. Accordingly, we silenced mitochondrial SQR to prevent mitochondrial H_2_S metabolism and evidenced that sFlt-1, but not sEng levels were suppressed by AP39. These observations suggest that mitochondrial H_2_S may have a dual regulatory effect in the regulation of sFlt-1 and sEng as either a ROS scavenging molecule and/or an enhancer of OXPHOS via SQR.

Antioxidants have largely proven not to be effective in the prevention of preeclampsia. However, it has been suggested that this inefficiency might occur due to antioxidants not reaching the correct intracellular location, such as the mitochondrion^52^. Consistent with other authors’ observations, our results evidenced mitochondrial dysfunction and oxidative stress in CSE/H_2_S defective pathways and restoration of the mitochondrial activity in the presence of AP39 (Fig. 2). It has been shown that H_2_S exerts cytoprotective effects against mitochondrial oxidative stress in diabetes^53,54^. Similarly, mitochondrial-targeted antioxidants have shown to prevent the upregulation of inflammatory markers TNF-α, UCP- and TRL9 in HUVEC^52^, prevented hypertension and improved pup and placental size in a reduced uterine perfusion pressure (RUPP) rat model of preeclampsia^55^. Collectively, these suggest that targeting H_2_S to the mitochondrion may prevent the upregulation of sFlt-1 by modulating the mitochondrial function and oxidant status.

Although mitochondrial-targeted H_2_S showed to reduce sFlt-1 levels in HUVEC with dysregulated mitochondrial SQR, levels of sEng were not statistically different. Similarly, mitoTempo did not exert effects on sEng production (Supplementary Fig. 5), suggesting that mitochondrial metabolism of H_2_S is required for sEng regulation. In the placenta, the matrix metallopeptidase 14 (MMP-14) has shown to directly mediates the release of sEng^56^. MMP-14 is a cleavage protease of placental endoglin that releases its extracellular domain into the maternal circulation to form sEng. . While our results suggest that regulation of sEng levels are associated with SQR-dependant H_2_S metabolism,the precise molecular mechanisms implicated in endoglin cleavage and its links to MMP-14 remain to be elucidated.

Here we provide first-time evidence demonstrating that mitochondrial metabolism of CSE-derived H_2_S regulates endothelial mitochondrial function, mitochondrial oxidative status, and production of antiangiogenic factors. We elucidate the mechanism for sFlt-1 and sEng production using a mitochondrial-targeted H_2_S donor and silencing the mitochondrial H_2_S metabolising enzyme, SQR. Our study suggests that reduced bioavailability of H_2_S within the mitochondrion reduces OXPHOS and promotes mitochondrial-specific ROS production while enhancing less efficient metabolic pathways. Thus, our findings propose that CSE-derived H_2_S may facilitate the endothelial mitochondrial metabolism and suppresse antiangiogenic factors production by either exerting antioxidant properties and/or stimulating the mitochondrial electron transport chain via SQR.

## Methods

### Cell culture and treatment

Human umbilical vein endothelial cells (HUVEC) pooled from different donors were obtained from PromoCell (Heidelberg, Germany) and cultured in standard endothelial cell growth media (EGM-2). To force mitochondrial metabolism, HUVEC were cultured in carbohydrate restrictive media, where glucose was replaced by galactose, matching the concentration of glucose present in cells culture media as previously described^23,32^.

HUVEC were treated with the slow-releasing mitochondrial-targeted H_2_S donor, AP39 (Cayman Chemicals, USA) at different concentrations for 24 h. AP39 is a slow-releasing H_2_S molecule (dithiolethione) coupled to a mitochondria-targeting motif, triphenyl phosphonium by an aliphatic linker, which allows the specific delivery of the H_2_S moiety into the mitochondrion^29^. Cell viability assays allowed to identify 100 nM as optimal concentration for treatments. In transfection assays, AP39 was administered at 100 nM after cell transfection. Cell supernatant was collected for ELISA analysis. All experiments were performed on third to fourth passage HUVEC.

### RNA interference

To compromise human CSE and mitochondrial SQR expression, we performed transfection of small-interfering RNA (siRNA) by electroporation using an Amaxa Nucleofector (Lonza, Switzerland), transfection program CA-167. siRNA for CSE were synthesized by IDT DNA technologies (Glasgow, UK) while nonsilencing siRNA and siRNA for SQR were purchased from Qiagen (Qiagen, Germany). Transfection was performed using P5 Primary Cell 4D-Nucleofector (Lonza, Switzerland), at 20 pmol for each siRNA and protocols provided by the manufactor. Knockdown of CSE and SQR in HUVEC was confirmed by Western blotting.

### Quantitative RT-PCR

HUVEC were transfected with CSE siRNA and cultured for 24 h. RNA was extracted using RNeasy mini-kit (Qiagen, Germany) and quantified using the Nanodrop ND 1000 spectrophotometer (NanoDrop Technologies Inc., USA). RNA was converted to cDNA using the EvoScript Universal cDNA Master (Roche Life Sciences, Switzerland) following manufacturers' guidelines. Gene expression of human CSE and YWHAZ were quantified by real-time PCR on a Lightcycler 480 (Roche Life Sciences, Switzerland) using the LightCycler 480 SYBR Green I Master and its specific primers. RT-PCR was performed using the following run conditions: Pre-incubation (1 cycle), amplification (45 cycles), melting curve (1 cycle), and cooling (1 cycle). Relative gene expression was calculated using the 2^−ΔΔCT^.

### Determination of intracellular H_2_S by fluorescence

H_2_S was measured in cells using the specific fluorescent probe Sulfidefluor-7 acetoxymethyl ester (SF7-AM) (Sigma Aldrich, USA) as previously described^57^. CSE siRNA transfected HUVEC were cultured for 24 h. Next, cells were incubated with 2.5 µM SF7-AM in culture media for 30 min. Cells were washed twice with warm PBS and the fluorescence was measured at 495 nm/519 nm. Images were recorded at 20X using a Nikon Eclipse Ti-E inverted microscope. Analysis were performed using Image J.

### Intracellular reactive oxygen species determination

Detection of intracellular reactive oxygen species was performed by fluorescence microscopy using the fluorescent dye DCFDA (Sigma Aldrich, USA). HUVEC transfected using CSE siRNA and plated in 24 well plates at a density of 5.0 × 10^4^ cells/well. After 24 h, cells were washed with warm PBS and incubated in 5 µM DCFDA probe in PBS for 30 min, protected from light. Following, cells were washed twice with warm PBS and the green fluorescence emitted was analysed at 529 nm using a Nikon Eclipse Ti-E inverted microscope using 20X objective lens. Images were recorded and analysed using Image J.

### Mitochondrial specific superoxide determination

Mitochondrial-specific superoxide was examined by fluorescence microscopy using the fluorescent dye MitoSOX Red (Sigma Aldrich, USA). Briefly, HUVEC were transfected with CSE siRNA and plated in 24 well plates at a density of 5.0 × 10^4^ cells/well. After 24 h, cells were washed with warm PBS and incubated in 5 µM MitoSOX probe in PBS for 30 min, protected from light. Cells were carefully washed with warm PBS twice and fluorescence emitted at 580 nm was recorded and analysed using a Nikon Eclipse Ti-E inverted microscope at 20 × as described previously^23^.

### Mitochondrial oxygen consumption

Detection of oxygen consumption rates were analysed in real-time using an XF24 Extracellular Flux Analyser (Agilent Seahorse). Briefly, CSE siRNA transfected HUVEC were plated at 4 × 10^4^ cells/well in V7 24 well plates (Agilent Seahorse) in standard growth media. After attached, cells were treated with AP39 at 100 nM for 24 h. Following, culture media was changed to a non-buffered DMEM media (glucose 10 nM, pyruvate 1 mM and glutamine 2 mM) to allow temperature and pH equilibrium. Oxygen consumption rates (OCR) were measured simultaneously three times to establish baseline measurements. Following, to evaluate mitochondrial function, oligomycin (1 mM) (Sigma Aldrich), carbonyl cyanide 4-(trifluoromethoxy)phenylhydrazone (FCCP) (0.5 mM) (Sigma Aldrich) and a mixture of rotenone and antimycin A (Rot/AntA) (1 mM) (Cayman Chemicals) were injected sequentially, to inhibit the ATP synthase, uncouple oxidative phosphorylation, and estimate non-mitochondrial respiration, respectively. This experiment measures six parameters of the mitochondrial function: basal oxygen consumption, ATP-linked oxygen consumption, proton leak, maximal oxygen consumption, reserve capacity, and non-mitochondrial oxygen consumption as described previously^23,58^. After the completion of the determinations, OCR measurements were normalized to protein content by the Bradford method.

### Aerobic glycolysis

CSE siRNA transfected HUVEC were plated at 4 × 10^4^ cells/well in V7 24 well plates (Agilent Seahorse) in standard growth media. After 24 h, cells were washed and media replaced to unbuffered media (1 mM pyruvate and 2 mM glutamine). Once in the instrument, extracellular acidification rates (ECAR) were measured simultaneously three times to establish a baseline rate for 3–5 min. To evaluate glycolytic response, cells were subjected to glucose (5.5 mM) (Sigma Aldrich), oligomycin (1 mM) (Sigma Aldrich) and 2-deoxi-glucose (2-DG) (100 mM) (Sigma Aldrich), to induce glycolysis, inhibit ATP synthase and estimate non-glycolytic acidification, respectively. This approach identified the glycolytic rate, glycolytic capacity and reserve and non-glycolytic acidification as described previously^23^. ECAR measurements were normalized to protein content by the Bradford method.

### ELISA

Quantification of sFlt-1 and sEng was performed using R&D Systems kits and performed according to the manufacturer’s specifications in cell supernatant.

### Western

Cell lysates (20 µg) were separated on 12% polyacrylamide gels with semidry transfer to PVDF membranes (Millipore, Billerica, MA). Membranes were blocked and and incubated overnight with either anti-CSE (Gamma Cystathionase Antibody, Proteintech # 12217-1-AP, dil 1:500) or anti-SQR (Anti-SQRDL antibody, Abcam # ab71978, dil 1:500). Bands were visualised using chemiluminiscense detection (GE Healthcare Life Sciences). Following imaging, the anti-CSE and anti-SQR antibodies were stripped using stripping buffer and probed overnight with either β-actin (Sigma Aldrich # A5441, dil 1:2,500) or VDAC (Anti-VDAC1/Porin antibody, Abcam # ab34726, dil 1:2,500). β-actin and VDAC were used as a loading controls. Images were cropped using Image J software. Full-length membranes are included in the supplementary section.

### Statistical analysis

Each test was performed in three independent technical replicates, with three to six independent biological replicates each. A power calculation was not performed. Shapiro–Wilk tests were performed to test the normal distribution of the data. Statistical analysis were performed using unpaired T-test (two groups) and ordinary one-way ANOVA (more than two groups) for parametric data and Mann–Whitney (two groups) and Kruskal Wallis test (more than two groups) for non-parametric data. Post-hoc analysis were carried out using either Bonferroni’s (parametric) or Dunn’s test (non-parametric). All bars and dot graphs show mean values ± SEM. Statistical analysis were performed using GraphPad Prism 8.1 software (GraphPad Software, La Jolla, CA; https://www.graphpad.com/scientific-software/prism/). Values of *p* < 0.05, *p* < 0.01, and *p* < 0.001 were considered statistically significant.

## Supplementary information

Supplementary information.
