# Supplementary material for: Bioenergetic effects of hydrogen sulfide suppress soluble Flt-1 and soluble endoglin in cystathionine gamma-lyase compromised endothelial cells
Source: Sci Rep. 2020 Sep 25;10:15810. doi: 10.1038/s41598-020-72371-2 (PMC7519095; doi:10.1038/s41598-020-72371-2)
Supplement: Supplementary file 1 — Supplementary information. [file 41598_2020_72371_MOESM1_ESM.pdf]

Supplementary Material

Bioenergetic effects of hydrogen sulfide suppress soluble Flt-1 and soluble endoglin in cystathionine gamma-lyase compromised endothelial cells

Lissette Carolina Sanchez-Aranguren[1], Shakil Ahmad[1], Irundika HK Dias[1] Faisal A. Alzahrani[2,3], Homira Rezai[2], Keqing Wang[1], and Asif Ahmed[1,2,3]*

[1]Aston Medical Research Institute, Aston Medical School, Birmingham, United Kingdom; [2]Mirzyme Therapeutics, Innovation Birmingham Campus, Faraday Wharf, Holt Street, Birmingham B7 4BB, United Kingdom. [3]Department of Biochemistry, ESC Research Unit, Faculty of Science, King Fahd Medical Research Center, King Abdulaziz University, Jeddah 21589, Saudi Arabia.

*Corresponding author:

Professor Asif Ahmed (asif.ahmed@mirzyme.com and @ProfAsifAhmed)

Mirzyme Therapeutics,

Innovation Birmingham Campus,

Faraday Wharf, Holt Street,

Birmingham B7 4BB,

United Kingdom

Supplementary Methods:

Cell viability assays

Cell viability was determined by MTT assay. HUVEC were plated at a density of 1.0 x 10^4 cell/well on 96-well plates, and grown for 24 h in EGM-2. Next, cells were treated with AP39 (0-1) µM for 24 h in EGM-2.

To assess the effect of glucose replacement by galactose, in other experiments, EGM-2 media was replaced by DMEM containing 10 mM (glucose or galactose, respectively), 4 mM L-glutamine, 1 mM pyruvate supplemented with 10% FBS for another 24 h. Following treatments, cells were exposed to 100 µL of MTT solution (1 mg/mL). Four hours later, formazan crystals were solubilized in dimethyl sulfoxide (DMSO). Absorbance was measured in a plate reader at 570 nm using a Tecan plate reader (Tecan, Switzerland).

Effects of mitoTempo on sFlt-1 and sEng release

First, to assess the effect of MitoTempo on cell viability, HUVEC were treated with MitoTempo (0.675-20) µM (Sigma Aldrich) for 24 h.

Further experiments were performed using 5 µM MitoTempo for 24 h in substrate-based approach using galactose instead of glucose as sole carbohydrate for cell culture. Cell supernatant was collected for further ELISA analysis. All experiments were performed on third to fourth passage HUVEC.

Quantification of sFlt-1 and sEng was performed using R&D Systems kits and performed according to the manufacturer’s specifications.

Supplementary Figures:

Supplementary Figure 1.

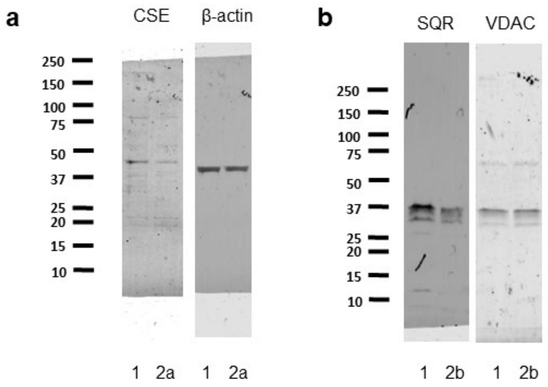

Supplementary Figure 1. Western blot full-lenght membranes. a) CSE and β-actin and b) SQR and VDAC. Key: 1: siRNA control (siCTL), 2a: siRNA for CSE (siCSE) and 2b: siRNA for SQR (siSQR) transfected HUVEC.

Supplementary Figure 2.

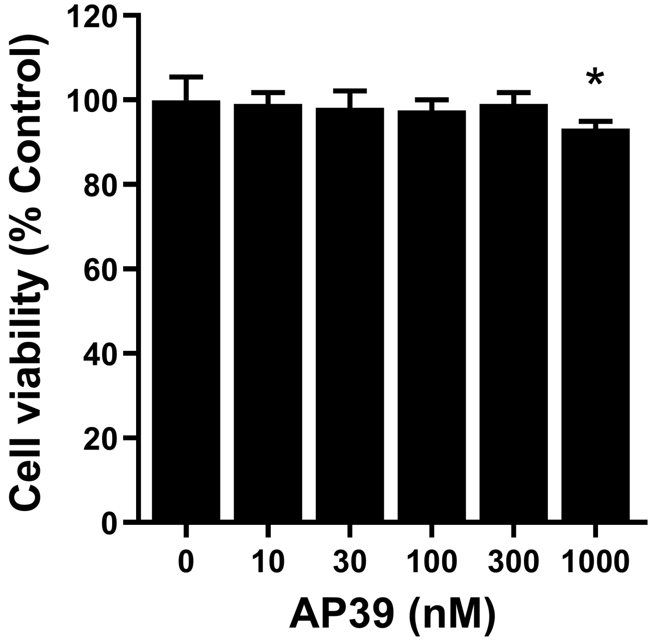

Supplementary Figure 2. Effect of mitochondrial-targeted H_2S donor, AP39 on cell viability. HUVEC were exposed to (10-1000) nM AP39 for 24 h. Values are expressed as means ± SEM, n=3. *P< 0.05 vs non-treated.

Supplementary Figure 3.

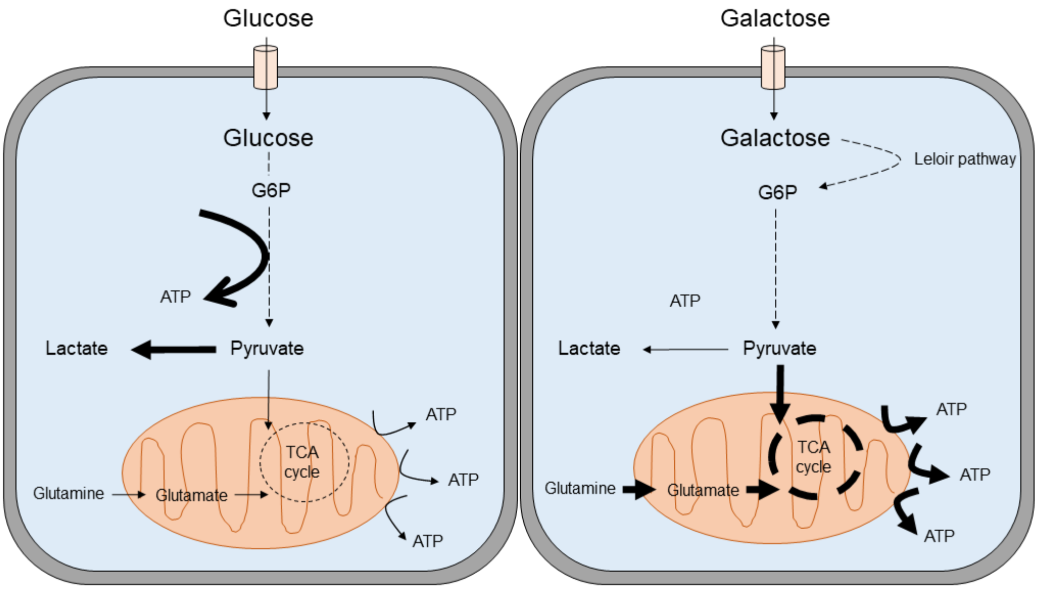

Supplementary Figure 3. Schematic representation of the replacement of glucose by galactose cells culture media. Galactose forces cells to rely on mitochondrial function and evidence the effects of molecules that modulate mitochondrial bioenergetics. As there is no catabolic pathway to metabolise galactose. Therefore, it is converted into glucose-6-phosphate in four steps that consume ATP (Leloir pathway). Glucose-6-phosphate is then metabolised via glycolysis yielding no net ATP production. This forces the cells to have an increased reliance on other OXPHOS substrates (such as pyruvate and glutamine) for energy[32]. The thickness of the arrows proportional to the underlying flux. This diagram was drawn using Microsoft PowerPoint .version 16.39.

Supplementary Figure 4.

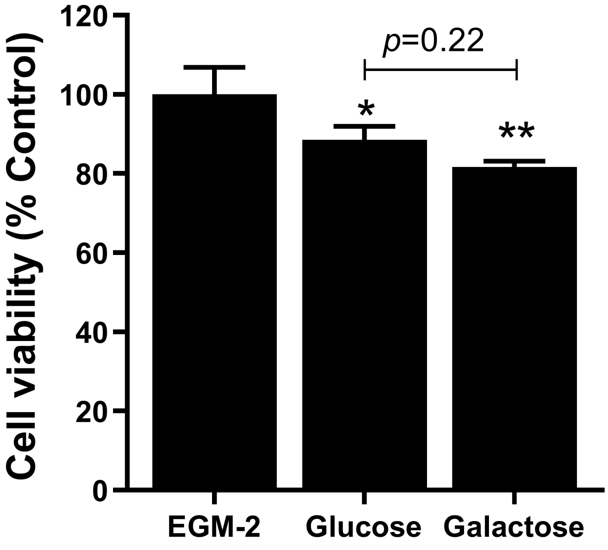

Supplementary Figure 4. Effect of carbohydrate replacement in cell culture media on cell viability. HUVEC standard growth media (EGM-2) was replaced by either glucose or galactose enriched media matching the same concentration of carbohydrate (10 mM) and cells were grown for 24 h.

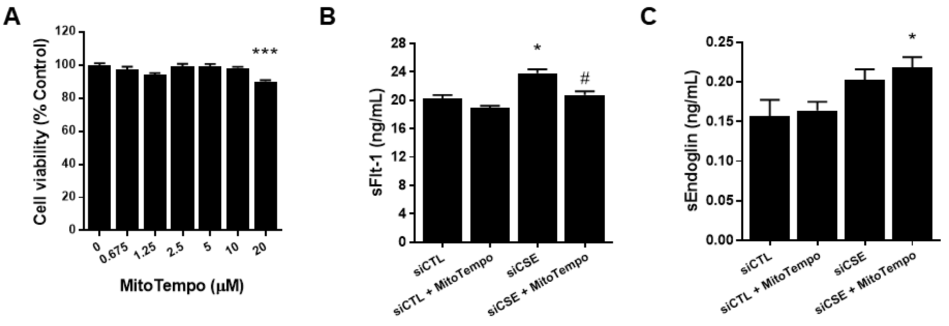

Supplementary Figure 5. Effect of MitoTempo on cell viability and anti-angiogenic factors production. (A) HUVEC were exposed to MitoTempo (0.675-20) μM for 24 h. Concentration of 5 μM was chosen for further experiments. (B) levels of sFlt-1 and (C) sEng were measured in culture media by ELISA. Values are expressed as mean ± SEM, n=3-6 experimental replicates. **P*< 0.05, ***P*< 0.01, ****P*< 0.001 vs siCTL. #*P*<0.05 vs siCSE.
